# *TSEN54* Gene-Related Pontocerebellar Hypoplasia Type 2 Could Mimic Dyskinetic Cerebral Palsy with Severe Psychomotor Retardation

**DOI:** 10.3389/fped.2018.00001

**Published:** 2018-01-23

**Authors:** Iliyana Hristova Pacheva, Tihomir Todorov, Ivan Ivanov, Desislava Tartova, Katerina Gaberova, Albena Todorova, Diana Dimitrova

**Affiliations:** ^1^Department of Pediatrics and Medical Genetics, Medical University – Plovdiv, Plovdiv, Bulgaria; ^2^St. George University Hospital, Plovdiv, Bulgaria; ^3^Genetic Medico-Diagnostic Laboratory “Genica”, Sofia, Bulgaria; ^4^Department of Medical Chemistry and Biochemistry, Medical University, Sofia, Bulgaria

**Keywords:** pontocerebellar hypoplasia, *TSEN54* gene, dyskinetic cerebral palsy, microcephaly, severe psychomotor retardation, epilepsy

## Abstract

Pontocerebellar hypoplasia (PCH) type 2 is a very rare autosomal recessive neurodegenerative disorder with prenatal onset that disrupts brain development. We present three patients (two siblings and one unrelated child) with PCH 2 linked to the most common mutation c.919G > T (p.Ala307Ser) in *TSEN54* gene. The disease started soon after birth with feeding difficulties, extrapyramidal symptoms, psychomotor retardation, progressive microcephaly. Two of the patients were diagnosed with dyskinetic cerebral palsy (CP) at first. Despite the neurodegenerative character of PCH 2, the absence of regression and even some developmental progress in few patients, might erroneously lead to the incorrect diagnosis of dyskinetic CP. Megacisterna magna on brain ultrasound makes the diagnosis of PCH 2 highly probable and should prompt further imaging with MRI. MRI findings of PCH are pivotal for the diagnosis. Genetic testing for the most common mutation in *TSEN54* gene should also be performed. Correct diagnosis of PCH 2 is essential not only for the prognosis of the patient, but also for prenatal diagnosis in future pregnancies. Knowledge of the clinical picture of PCH 2 will lead to correct and timely diagnosis. Advanced neuroimaging procedures and molecular genetic techniques provide valuable tools for prompt diagnosis of rare, but clinically important, neurogenetic imitators of CP.

## Introduction

Pontocerebellar hypoplasia (PCH) is a group of very rare autosomal recessive neurodegenerative disorders with prenatal onset that disrupt brain development. During the past two decades, the spectrum and classification of PCH has expanded significantly. There were only two types in 1990s, now the classification of PCH includes 10 types with characteristic clinical, imaging and genetic features (1). Type 2 is the most common and the first genetically described PCH by Barth, although its frequency is rather low, 1 per 200,000 people (1–3). There have been less than 100 published cases in the literature so far, none of them from Bulgaria (2–15).

Pontocerebellar hypoplasia type 2 (PCH 2) is associated with mutations in several genes: *TSEN54, TSEN2*, and *TSEN34* (1, 3, 4). They are vital for neuronal development, regulating the splicing of tRNA and consequently affecting protein synthesis. The most common mutation that causes PCH 2 is in *TSEN54* gene, localized on chromosome 17 (3, 4).

Pontocerebellar hypoplasia 2 presents in the first months of life, or soon after birth, as feeding difficulties due to swallowing incoordination; regurgitations, vomiting, failure to thrive, respiratory difficulties or apneas, sleep disorders like difficulty in staying asleep or excessive sleepiness. Extrapyramidal symptoms are also prominent in the clinical picture. Microcephaly can be diagnosed at birth, but usually becomes obvious in the next few months due to the progressive disease course. Neurodevelopment is severely affected. Sometimes infants can have normal neurodevelopment during the first 2 months, which stops or sometimes regresses afterward.

We present three cases with *TSEN54* gene-related PCH 2 with dyskinetic cerebral palsy (CP)-like clinical picture. Two of them are siblings (case 1 and case 2), while case 3 is unrelated to them (Table 1).

**Table 1:** Characteristics of pontocerebellar hypoplasia (PCH) 2 in our patients.

Typical features	Case 1	Case 2	Case 3
Onset of symptoms	2–3rd month	2nd month	3rd month
Age of diagnosis	3 months	4 years 3 months	8 months
Family history	Yes	Yes	No
Intrauterine growth retardation	No	No	Yes
Green amniotic fluid during delivery	Yes	Yes	Yes
Microcephaly at diagnosis (head circumference)	Suboptimal head growth (−1.9 SD)	Severe (−6.2 SD)	Yes (−3.75 SD)
Psychomotor developmental quotient at diagnosis	35	<20	25
Muscle tone	Spastic	Changeable	Changeable
Choreoathetotic movements	Yes	Yes	Yes
Dystonic attacks	No	Yes	Yes
Epilepsy	Probable epileptic spasms	Generalized tonic–clonic, complex partial seizures, probable epileptic spasms, status epilepticus	1 suspicious clonic seizure
Interictal epileptiform discharges on scalp EEG	Generalized	Multifocal and generalized	No
Gastrooesophageal reflux signs (regurgitations, vomiting, irritability during or after feeding)	Yes	Yes	Yes
Sleep disorders (difficulty staying asleep, frequent awakening with crying)	Yes	Yes	Yes
Failure to thrive	No	Yes	Transient
Enlarged cisterna magna on brain ultrasound scan	Yes	Yes	Yes
Brain MRI (age)	–	PCH—dragonfly-like cerebellar pattern (2 years); progressive cerebellar atrophy (4 years)	PCH—dragonfly-like cerebellar pattern (8 months)
Fundoscopy	Normal	Normal	Normal
Preceding diagnosis of dyskinetic cerebral palsy	No	Yes	Yes
*TSEN54* gene mutation	Homozygous missence mutation c.919G > T (p.Ala307Ser)	Homozygous missence mutation c.919G > T (p.Ala307Ser)	Homozygous missence mutation c.919G > T (p.Ala307Ser)

## Case Presentation

### Case 1

A 3-month-old male infant, born by cesarean section at full term to nonconsanguineous Bulgarian parents, with normal Apgar score. The amniotic fluid was noted to be green but there were no other symptoms of perinatal asphyxia. His birth weight, length, and head circumference were within the normal ranges. At the age of 3 months, he started having feeding problems (unconsolable crying after feeds, regurgitations). Movement disorders like dystonic postures and spontaneous Moro reflex were also noticed. Neurodevelopmental delay was registered: visual pursuit of objects and faces was inconstant; he started smiling at 3 months of age and then stopped. He was not cooing.

#### Family History

His 4-year-old sister was diagnosed with dyskinetic CP because of microcephaly, profound psychomotor delay, dyskinesias, and epilepsy.

Physical examination of the patient at the age of 3 months was normal. Only head circumference showed low growth rate (−1.9 SD). Neurologic examination revealed decreased tone of neck muscles with absent head control, spasticity of the extremities, tendon hyperreflexia, and persistence of grasp (Robinson) reflex and automatic crawling. General movements were not fluent, but jerky and/or stiff. Dystonic postures were frequent. There was moderate psychomotor delay with developmental quotient (DQ) of 35.

##### Laboratory, Neurophysiological, and Imaging Investigations

A complete blood cell count, biochemistry and arterial blood gases were within the reference ranges. No abnormal organic acids were found in the urine. Plasma acylcarnitine and amino acid profiles were normal. Serology testing for congenital infections (Cytomegalovirus, Rubella, Toxoplasma gondii) and Ebstein Barr virus, was negative. Sleep EEGs (lasting about 30 min with no seizure-like events) showed occasional primary generalized single spike-and-slow-wave complexes.

**Figure 1:**
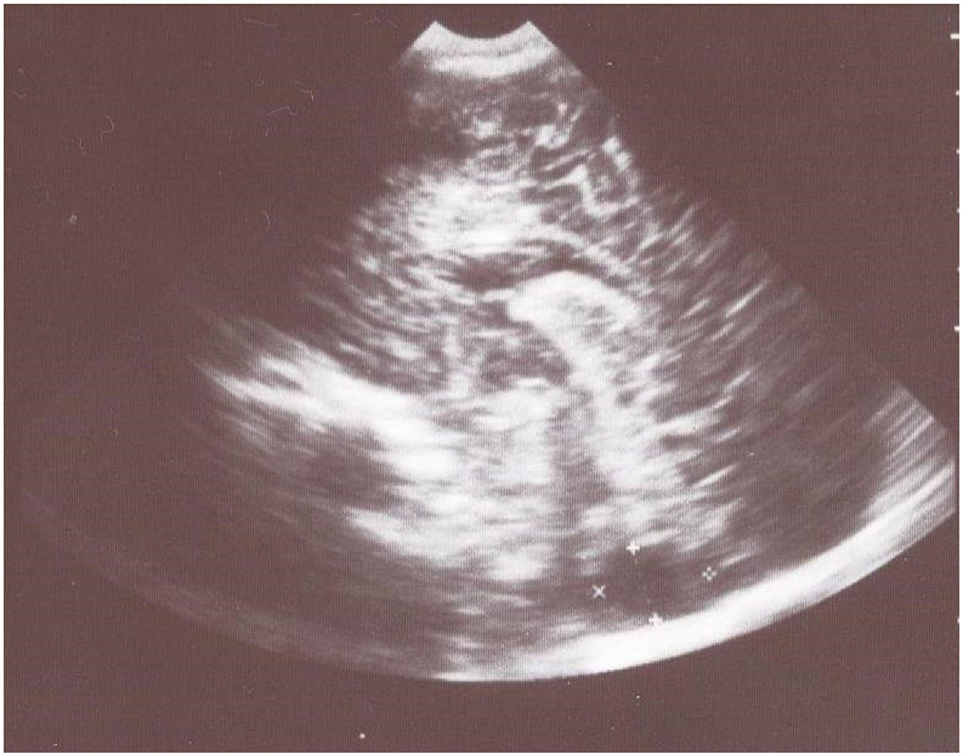
Transfontanelle sonography shows large cyst-like cisterna magna that suggested cerebellar hypoplasia and slightly dilated frontal horns of the lateral ventricles.

Transfontanelle sonography showed slightly dilated lateral ventricles and large cyst-like cisterna magna that suggested cerebellar hypoplasia (Figure 1).

Pontocerebellar hypoplasia 2 was suspected and confirmed by DNA sequencing of the *TSEN54* gene: a homozygous missence mutation c.919G > T (p.Ala307Ser) was found.

#### Follow-up

At the age of 1 year 6 months, a little progress in psychomotor development was observed and DQ was below 30. He was smiling and cooing. He had started to follow objects and to roll occasionally. Spastic quadriparesis with extrapyramidal features persisted. Hyperkinesias occurred less frequent. Feeding problems decreased with time.

### Case 2 (Sibling of Patient 1)

A 4-year-old girl from an uneventful pregnancy. Apgar score at birth was optimal, there were no symptoms of perinatal asphyxia, but the neonatal discharge letter described the amniotic fluid as green and placental calcification (without additional investigations).

Her disease started in the first 2 months after birth with developmental delay: there was no head control and no visual pursuit of objects at 2 months of age. There was also sucking discoordination and slow head growth. Abnormal movements like epileptic spasms started during the third month. Interictal sleep EEG demonstrated background slowing without hypsarrhythmia. Prolonged EEG was not recorded. Transfontanelle sonography showed slightly dilated lateral ventricles and a megacisterna magna. She was treated with adrenocorticotropic hormone and valproic acid with no effect. At the age of 15 months, the patient had generalized tonic–clonic status epilepticus. Sleep interictal EEG showed disorganized background activity and generalized spike-wave discharges. Her epilepsy was resistant to valproate and clonazepam.

MRI at 2 years of age demonstrated hypoplastic pons and cerebellum, and wide ventricular and subarachnoid spaces. PCH was suggested by the neuroradiologist, but was not pursued further by the clinicians.

There was no progress in her psychomotor development after 2 months of age. She had sleep disorder, frequent hyperkinesias, and also dystonic attacks. Rare apneas were also observed by the parents, as well as impaired swallowing with choking when drinking fluids. Secondary generalized seizures in clusters of 5–10, lasting 1–2 min persisted with frequency one cluster per 10 days, and levetiracetam had been started.

At the age of 4 years, after the clinical diagnosis of her brother had been made, the girl was admitted to our department for reevaluation.

Physical examination revealed severe microcephaly: head circumference 41 cm (−6.2 SD) and failure to thrive: weight 10 kg, length 99 cm.

Neurologic examination demonstrated quadriparesis with changeable muscle tone, but prevailing spasticity, frequent choreoathetoid movements and dystonic C-like posturing, exaggerated tendon reflexes with ankle clonuses, bilateral Babinski sign, absent visual fixation and no acousticopalpebral reflex. Neuropsychological assessment established profound psychomotor retardation (DQ < 20).

**Figure 2:**
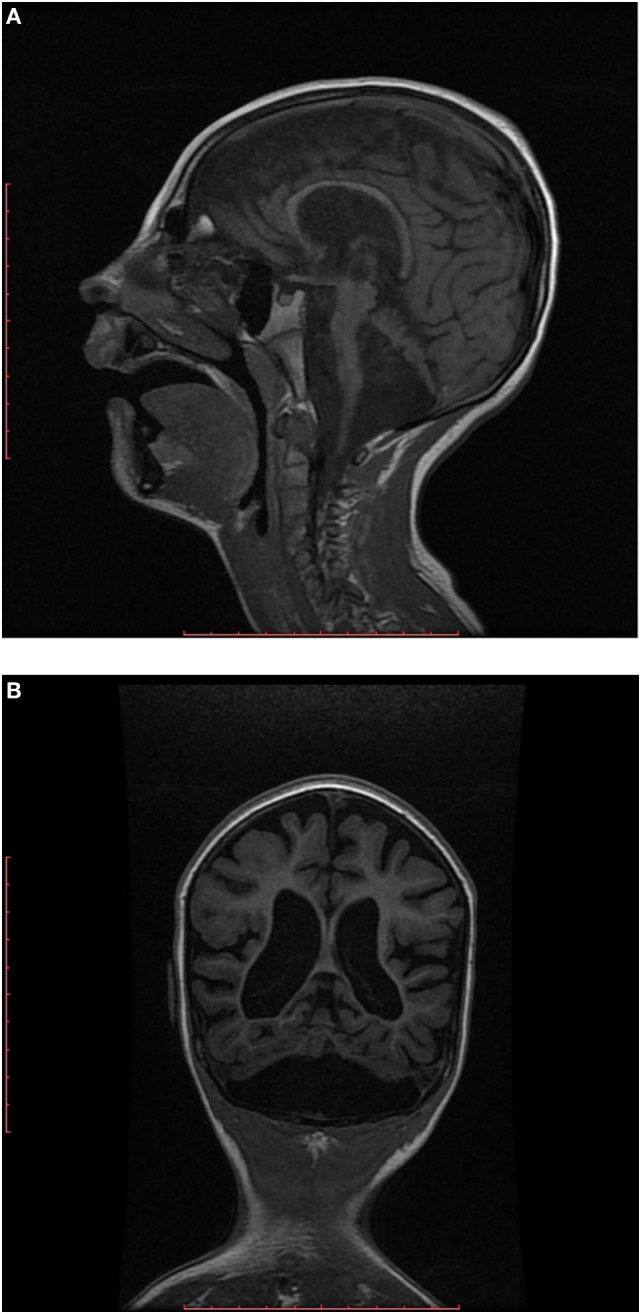
**(A)** Sagittal T1-weighted image shows hypoplastic cerebellum (cerebellar hemispheres more affected than vermis) associated with a small pons. **(B)** Coronal T1-weighted image shows markedly hypoplastic cerebellar hemispheres (dragonfly-like cerebellar pattern), with dilatation of the cisterna magna inferiorly and also cortical and cerebral atrophy.

EEG showed disorganized background, and generalized paroxysmal activity of fast spike-slow wave complexes. MRI demonstrated generalized brain atrophy with marked cerebellar hypoplasia, dragonfly-like cerebellar pattern, and hypoplasia of pons (Figures 2A,B).

DNA analysis confirmed PCH 2 by finding the same (as in her brother) homozygous missense mutation c.919G > T (p.Ala307Ser) in *TSEN54* gene. Parents were found to be heterozygous carriers.

#### Follow-up

During the next year, there was no change in her condition and seizure frequency. Levetiracetam was discontinued.

### Case 3

A 8-month-old male infant, born to nonconsanguineous Bulgarian parents, after an uneventful pregnancy and delivery at 38th gestational week, with optimal Apgar scores, but green-colored amniotic fluid. He was small for gestational age (weight 2.2 kg, length 46 cm, head circumference 32 cm). There were no symptoms of perinatal asphyxia or respiratory problems in the neonatal period. Neurodevelopmental delay was noted around 3–4 months of age: he could not grasp objects and could not roll and sit; his visual pursuit was not constant. Episodes of irritability with crying and turning his head backwards, usually after feeding, as well as frequent regurgitations and choreoathetoid movements started at 5–6 months of age. At that time diagnosis of CP was suggested and rehabilitation was recommended. At 8 months of age he was admitted to our department with acute respiratory infection, increased irritability and extensor spasms-like jerky movements.

Physical examination revealed microcephaly: head circumference 41 cm (−3.75 SD).

Neurological findings were changeable muscle tone with frequent increases of axial extensor muscle tone, exaggerated tendon reflexes and bilateral Babinski sign, persistent primitive reflexes (first phase of Moro, automatic walking, and grasping). There were choreoathetoid hyperkinesias. DQ was 25. He could fix, but could not follow objects, he rarely smiled.

#### Laboratory, Electrophysiological, and Imaging Investigations

Complete blood cell count was within reference ranges, biochemistry showed elevated creatine kinase to 1,388.0 U/l. Arterial blood gases revealed metabolic lactic acidosis (pH 7.28) with elevated lactate to 6.7 mmol/l during the acute infection. Lactate decreased to 2.9 mmol/l and metabolic acidosis disappeared after recovery. CSF glucose, protein and cells; organic acids in urine; the profiles of plasma amino acids and acylcarnitines were within the reference ranges. Serological investigations for common congenital infections were negative.

Several “interictal” sleep EEGs, lasting about 30 min, were normal. Transfontanelle sonography showed cystically dilated cisterna magna and slightly dilated lateral ventricles. MRI demonstrated PCH with dragonfly-like cerebellar pattern, and moderately dilated lateral ventricles.

Our clinical diagnosis was PCH. We suspected PCH type 6 initially because of the transient lactic acidosis, but PCH 2 was also considered possible. DNA analysis for the common mutation in PCH 2 established the final diagnosis: *TSEN54* gene-related PCH 2. No DNA analysis was performed on the parents as they refused.

#### Follow Up

One year after the diagnosis there was no worsening, but some mild improvement in his psychomotor development. Hyperkinesias were less frequent. He had achieved head control and had started to follow objects constantly and to smile when addressed, to recognize his mother, to respond to sounds and to coo. However, he could not roll and sit, and he could not grasp objects. DQ remained below 30. Spastic quadriparesis persisted. He could eat only mashed food and had regurgitations occasionally.

## Discussion

The PCH 2 diagnosis in all three cases was considered because of a combination of the following findings: abnormal muscle tone, extrapyramidal hyperkinesias, severe delay in psychomotor development, progressive microcephly and MRI data of pontocerebellar atrophy with dragonfly-like cerebellar pattern. We excluded PCH types 4 and 5 (which are also *TSEN54* gene related) because our patients had no problems in the neonatal period, no joint contractures specific for PCH type 4, and no fetal seizure-like movements typical for PCH type 5 (14).

The most specific clinical syndrome of PCH 2 is the extrapyramidal one. It presents with changeable muscle tone, dystonic postures, choreoathetoid hyperkinesias that start during early infancy. Thus PCH 2 could mimic dyskinetic CP and could remain unrecognized. Improvement of extrapyramidal signs later in the course of the disease, as seen in our cases 1 and 3, is unusual. Dystonic attacks (twisted C-shape body posture) last more than 5 min, do not correlate with paroxysmal EEG activity and stop during sleep. In half of the patients these attacks cease with age (3). In our series, case 2 had typical dystonic attacks which did not improve with time, while case 3 had transient dystonic crises provoked by a respiratory infection and which disappeared after recovery. Good response of extrapyramidal symptoms to l-DOPA has been reported (16).

Another characteristic feature of PCH 2 is progressive microcephaly, which becomes obvious after 2–3 months of age, as seen in cases 1 and 2. In all patients, microcephaly progresses markedly with age. Microcephaly can also occur in CP as a result of severe hypoxic–ischemic encephalopathy and could lead to underdiagnosis of PCH 2.

Neurodevelopment is severely affected and usually stops at the age of 2–3 months. As PCH 2 is a neurodegenerative disease, neurodevelopmental progress and achievement of some milestones, as seen in case 3, is unusual. Sánchez-Albisua et al. also found that children with this *TSEN54* mutation could achieve some neurodevelopmental abilities like rolling, sitting without support, staying on all four limbs, grasping and holding objects, social smiling and recognizing familial people, pronouncing certain sounds and responding to praise (3). Central visual failure and lack of head control are major neurological signs, but among our cases patient 3 could fix and follow objects, i.e., had preserved central vision. This has been observed in other patients (3). These data illustrate the phenotype heterogeneity, especially in developmental milestones, in a genetically homogeneous subgroup of PCH 2 with the same mutation.

Epilepsy occurs in more than half of the patients with PCH 2 and presents with variable seizure types (3, 14, 15). Typical EEG abnormalities are background slowing and multifocal epileptiform activity. There are no reports of hypsarrhythmia in the literature, despite observations of epileptic spasms with typical ictal EEG correlation (15). Two of our patients had movement paroxysms suggestive of epileptic spasms, but without ictal EEG proof (no ictal EEG record), so extrapyramydal origin remained an alternative explanation. Epilepsy is usually pharmacoresistant, as in case 2 (3).

Impaired swallowing, a result of buccopharyngeal incoordination, is a major diagnostic criterion (17). It occurred in all of our patients at the beginning of the disease. They also had frequent regurgitations, irritability associated with feeding, suggestive of gastroesophageal reflux. Symptoms of gastroesophageal reflux could be added to the diagnostic criteria for buccopharyngeal incoordination in PCH 2 (14).

Sleep disorders are reported by nearly all parents as difficulty falling asleep, frequent awakening, irritability, crying, frequent abnormal postures. Apneas occur in more than half of the patients, but were registered in only one of our cases (case 2). As no polysomnography and pulse oximetry monitoring were performed we might have underdiagnosed sleep apnea in our patients.

All our cases had meconium-stained amniotic fluid during delivery, but Apgar scores were normal and there were no other signs of perinatal asphyxia or neonatal encephalopathy. Neonatal problems are reported in more than 60% of patients—feeding difficulties, respiratory disorders and/or apnea, abnormal muscle tone, jitterness (3). Birth weight was under the 10th percentile only in case 3.

Patient 3 had elevated creatine kinase, which is accepted as a minor diagnostic criterion by Barth et al. (17).

Transfontanelle sonography revealed enlarged cisterna magna, a sign of cerebellar hypoplasia, in all of our cases. Sonography is easy to perform in infants with neurological problems and should be considered as the first screening imaging method. We propose that the combination of “dyskinetic CP” with severe neurodevelopmental delay, and ultrasound data of megacisterna magna is suggestive for PCH and should prompt MRI.

The key diagnostic finding for PCH 2 is the MRI data of hypoplasia of cerebellum and pons. Cerebellar hemispheres are so grossly abnormal that the dragonfly pattern might be considered relatively specific for PCH 2 and should not be disregarded as in case 2, and should alert clinicians to this diagnostic possibility.

Cerebellar hypoplasia is not an isolated symptom in PCH 2. Atrophy of the cerebral hemispheres is also found and may erroneously be interpreted as a result of hypoxic–ischemic pre- and/or perinatal brain injury. MR tractography shows almost full absence of transverse pontine crossing fibers and this could be additional finding in favor of PCH 2 (12).

Differential diagnosis of *TSEN54* gene-related PCH 2 includes variable diseases, many of whom genetic in origin, but also dyskinetic CP (Table 2). Although individually rare, neurogenetic disorders are collectively common and could imitate CP. The diagnosis of CP should be doubted if history and MRI do not correlate with perinatal brain injury and there is family history of “CP,” especially the dyskinetic type (18). Many additional investigations are required for children with “dyskinetic CP” to exclude all other diseases. MRI can further focus testing for specific disorders, using chromosomal microarray, targeted gene sequencing and whole-exome sequencing (18, 19).

**Table 2:** Differential diagnosis of pontocerebellar hypoplasia type 2.

Cerebral palsy—dyskinetic type
Nonprogressive cerebellar malformations (Dandy-Walker, Joubert syndrome)
Cerebellar hypoplasia
Cerebellar dysplasia
Cerebellocerebral atrophy (SEPSECS gene)
X-linked CASK mutation
Congenital muscular dystrophy
Congenital CMV infection
Neurotransmitter disorders/Dopa responsive dystonia
Congenital disorders of glycosylation
Other inborn errors of metabolism—creatine deficiency, Lesch–Nyhan, organic acidurias, glucose transporter type 1 deficiency, serine deficiency
Mitochondrial diseases
Leucodystrophies
Ceroid lipofuscinosis
Infantile neuroaxonal dystrophy

The first diagnosis of case 2 and case 3 was dyskinetic CP. This fact implies that PCH 2 may be underdiagnosed in our country, assigning the cases to the broad entity of CP. The same situation might be in many other countries. Despite the neurodegenerative character of the disease and only slight increase in volume of infratentorial structures postnatally, very few patients demonstrate regression in their developmental milestones (20). The absence of regression in our cases supports the hypothesis of Sánchez-Albisua et al., that there is an early onset degeneration which thereafter stabilizes, so that patients could achieve some developmental progress (3). The absence of regression, even more the possibility for some developmental progress, might erroneously lead to incorrect diagnosis of dyskinetic CP. The latter is thought to be a result of perinatal asphyxia in about 70% (although birth asphyxia comprises 8–10% of etiologic causes for all types of CP) (21, 22). Genetic diseases could present as dyskinetic CP and in the past decades of the last century a recurrence risk of athetoid CP was estimated to be up to 10% (21). More recent study suggested 1.6% of recurrence of athetoid CP, combining data with previous studies (23). We could speculate that the decrease in recurrence rate could be due to the improvement of etiologic diagnosis of CP and excluding some of the genetic progressive diseases from the group of “dyskinetic CP.” Advanced neuroimaging procedures and molecular genetic techniques provide valuable tools for prompt diagnosis of rare but clinically important neurogenetic imitators of CP.

It is important to differentiate PCH 2 from dyskinetic CP, not only for the prognosis of the patients, but also for prenatal diagnosis in future pregnancies. Sánchez-Albisua et al. report seven families with two affected children, probably because of misdiagnosis of the first child (3). The authors point that there was a decrease in the age at diagnosis in the recent years in Germany and Switzerland—average 6 months for children born after 2008, compared to 5 years for children born before 2003 (3). This is probably the result of an improved diagnostic process. Pediatricians and pediatric neurologists should be aquainted with PCH 2.

All our cases with PCH 2 had the same homozygous missense mutation (p.A307S) and the parents were not consanguineous. Therefore, this mutation might be the most common for PCH 2 in Bulgaria as in other European countries (3, 4, 6, 7). That is why we could recommend patients with clinical diagnosis of PCH 2 to be tested firstly for this mutation.

## Conclusion

Pontocerebellar hypoplasia 2 could mimic dyskinetic CP, associated with severe psychomotor retardation and progressive microcephaly. Megacisterna magna on brain ultrasound scan in an infant with “dyskinetic CP” and psychomotor delay makes the diagnosis of PCH 2 highly probable and requires MRI. MRI data of PCH is the key diagnostic criterion. Establishing the same homozygous missense mutation (p.A307S) for PCH 2 in three Bulgarian children from two unrelated families could suggest that it is as common in Bulgaria as in other Caucasian populations. We can recommend all patients with PCH 2 to be tested firstly for this mutation of *TSEN 54* gene. Acquaintance with the clinical picture of PCH 2 will lead to correct and timely diagnosis.

## Ethics Statement

The study was performed in accordance with the Declaration of Helsinki principles and ethical standards. The study was approved by Institutional ethics committee of Medical University – Plovdiv. All parents of the patients gave written informed consent for publication of the case reports in a journal.

## Author Contributions

IP contributed to conception and design, acquisition, analysis, and interpretation of data; drafted the manuscript; gave final approval; agreed to be accountable for all aspects of the work. TT substantially contributed to conception, analysis of data; critically revised the manuscript for important intellectual content; gave final approval; agreed to be accountable for all aspects of the work. II substantially contributed to conception, analysis, and interpretation of data; critically revised the manuscript for important intellectual content; gave final approval; agreed to be accountable for all aspects of the work. DT and KG contributed to acquisition, analysis, and interpretation of data; critically revised the manuscript; gave final approval; agreed to be accountable for all aspects of the work. AT and DD contributed to analyses and interpretation of the data; critically revised the manuscript for important intellectual content; gave final approval; agreed to be accountable for all aspects of the work.

## Conflict of Interest Statement

The authors declare that the research was conducted in the absence of any commercial or financial relationships that could be construed as a potential conflict of interest.
